# The Perceived Quality of Life of Older People in Spain Who Care for Grandchildren and Related Variables: A Mixed Methods Study

**DOI:** 10.3390/healthcare12101037

**Published:** 2024-05-16

**Authors:** Sofía García-Sanjuán, Ana Isabel Gutiérrez-García, María José Cabañero-Martínez, Juan Miguel Aguilar-Sánchez, María Carmen Rocamora-Rodríguez, Silvia Escribano

**Affiliations:** 1 Department of Nursing, Faculty of Health Sciences, University of Alicante, Carretera de San Vicente del Raspeig, s/n, 03690 Alicante, Spainmcarmen.rocamora@ua.es (M.C.R.-R.); silvia.escribano@ua.es (S.E.); 2 Torrevieja Health Department, Guardamar del Segura Health Centre, 03140 Alicante, Spain

**Keywords:** grandparents, grandchildren, caring, quality of life, parenting, mixed methods design

## Abstract

Grandparental care of grandchildren is a prevalent social phenomenon. This study explores the perceptions of health-related quality of life of grandparents caring for their grandchildren. A mixed methods design was developed. In the first phase, participants were interviewed using a baseline questionnaire. The second phase consisted of focus groups with 19 of the 100 participants in the quantitative phase. The scores obtained from the quantitative analysis are in line with the qualitative data; they reflect that grandparent carers who are more involved in the care of their grandchildren have more symptoms of depression and stress and have poorer perceptions of physical health-related quality of life. What may at first appear to be a positive aspect, keeping grandparent carers active, can become negative when it comes to shared care and when the grandparents’ willingness to provide care is abused.

## 1. Introduction

The family, a historical and dynamic global institution made up of a series of interconnected networks, plays a key role in the development of individuals and their acquisition of society’s predominant values [1]. It spans the entire life cycle, providing a stable and supportive frame of reference and creating an environment for providing and receiving care. In the course of history, however, the family, as well as the construct itself, has undergone various changes both in terms of its organisation and the scope of its relationships. This is something that has been experienced by all societies at varying speeds and intensities [1,2]. 

Most 20th century Spanish families were rooted in a structure of patriarchy and marital durability tied to the Catholic faith [3] However, this traditional model has rapidly evolved and adapted to become a contemporary family model. In this model, intergenerational ties take on particular importance. This is mainly due to the involvement of grandparents in the upbringing or care of their grandchildren, in either a central or peripheral role, with the term “grandparenting” having been coined to describe this process. Grandparenting is not one single condition or situation but a dynamic relationship linking two non-continuous generations (grandparents and grandchildren) [4].

The causes of this shift in the family model towards “grandparenting” are many and complex, and they are linked to various sociodemographic factors [5]. As a result of these changes, older people have become more involved in decision making within the family system and have consequently taken on greater responsibility. This has led to more active involvement in the tasks associated with caring for the youngest family members [6].

There is a significant impact on the health-related quality of life of grandparents caring for their grandchildren. This can be influenced by several variables related to the provision of care, including the intensity of care, i.e., the number of hours they have to care for their grandchildren. So-called “custodial care” (more than 12 h a day) appears to have a negative impact on grandparents’ health. It is associated with higher levels of stress and depression, psychological distress and the presence of psychosomatic symptoms, as well as the onset or exacerbation of certain chronic conditions, such as diabetes or hypertension [7,8]. In contrast, supplementary or occasional care of between 15 and 20 h per week appears to have health benefits for grandparents in terms of maintaining greater physical mobility, improved life satisfaction and lower levels of depressive symptoms [8,9].

Providing care also has a bearing on grandparents’ work and financial situation. Grandparents who are less well-off or work fewer hours are more likely to be at risk of depression [10]. Another important variable is the relationship between parents and grandparents. This is among the most influential and can lead to clashes over authority and love for the children. It should also be borne in mind that the age of both grandparents and grandchildren is a variable that directly affects the health of the grandparents themselves [11]. As the age of the grandchild increases, grandparents report higher rates of depression and poorer quality of life [12].

Compared to the rest of Europe, Spain is one of the countries where households are formed by family ties with a more deeply rooted family culture [13] and where intergenerational relations continue to be an essential pillar of social welfare. In this context, spending on social protection is among the lowest in the European Union. This directly affects the family environment and is evidence of the limited commitment of the Spanish government compared to the European average and the countries with the highest spending in this area [14].

The Survey of Health, Ageing and Retirement in Europe (SHARE) [15] shows that 22.7% of grandparents take care of their grandchildren. It also highlights that Spain is the country with the lowest percentage of grandparent carers compared to the rest of the European Union. However, those who do provide care do so with greater intensity [15]. 

The work of grandparents is not acknowledged in Spain, unlike in countries such as Italy, Hungary or Germany, where there are tax incentives [16]. This may be because the intergenerational bond between grandchildren and grandparents is still very strong and, on the whole, the grandparent–grandchild relationship seems to have a positive impact on grandparents [4]. Most of the studies carried out in Spain have focused on analysing physical, economic and/or emotional variables and their influence on the health of grandparents [4,5]. They also look at the impact of grandparental care on their grandchildren’s education, on the formation of intergenerational relationships and on the specific factors that make older people more inclined to care for grandchildren. However, they do not examine grandparents’ experiences of taking on these duties [17,18]. 

The debate on the impact of grandparental care on grandparents’ health is still ongoing. Therefore, this study uses a mixed methods approach to explore grandparents’ experiences of caring for grandchildren and how this activity affects their perceived health-related quality of life. 

### Objective

To explore the perceived health-related quality of life of grandparents who care for their grandchildren. 

## 2. Materials and Methods

A two-phase mixed methods design was developed. The first phase was an observational/descriptive study. Participants were interviewed using a baseline questionnaire. The second phase involved focus groups [19] made up of grandparents who had taken part in the first phase of the study.

### 2.1. Phase 1

#### 2.1.1. Participants

The study population was grandparent carers in the Torrevieja Health Area (Guardamar del Segura Health Centre) and the Elda Health Area (Acacias Health Centre). The inclusion criteria were as follows: (1) people attending routine check-ups with the nurse at the health centres in these health areas, (2) people who spoke and understood Spanish, (3) people who wished to volunteer for this project, (4) people who were not frail or had functional or sensory limitations, and (5) people who cared for grandchildren without any degree of dependency or debilitating illness. Older adults were excluded if they had cognitive or other impairments that would interfere with comprehension and the ability to complete the questionnaire. 

The sample was systematically recruited throughout the data collection period between September and December 2021, selecting all participants who met the inclusion criteria.

#### 2.1.2. Variables and Instruments

A data collection booklet containing the following variables and reporting instruments was prepared for the initial assessment: 

The SF-12 Quality of Life Questionnaire: This questionnaire measures health-related quality of life. The SF-12 is a subset of 12 items from the SF-36 [20], measuring eight dimensions of positive and negative physical and mental health status (physical functioning, physical role, bodily pain, mental health, general health, vitality, social functioning and emotional role). It has adequate psychometric properties [21].

The abbreviated Yesavage Geriatric Depression Scale [22] is a 15-item self-administered dichotomous (yes/no) scale specifically designed for the older population. It has adequate psychometric properties [23].

The Perceived Stress Scale (Cohen) [24] is a self-report instrument assessing the level of perceived stress over the previous month. It consists of 14 items that are answered on a five-point Likert scale. The Spanish version [24] showed adequate psychometric properties.

Anxiety subscale of Goldberg’s Anxiety and Depression Scale (GADS): This was developed in 1988 from a modified version of the Psychiatric Assessment Schedule [25] The Spanish version was validated by Montón et al. [26] and consists of two subscales of nine items each: one for anxiety (items 1–9) and the other for depression (items 10–18). All items are dichotomous (yes/no) and have adequate psychometric properties.

As well as these instruments, the following sociodemographic variables were collected: sex, age, marital status (categorical variable: single, married or cohabiting, separated or divorced, widowed), number of children and number of grandchildren. Variables were also collected on the time spent caring for their grandchildren and how long they had been doing so, with caring for their grandchildren being defined as all the time spent with them. 

#### 2.1.3. Data Collection

The sample selection process and the request for consent to participate in the study were carried out by health professionals in their health centres and consulting rooms. These practitioners helped make contact with people who met the inclusion criteria, once the sociodemographic data questionnaire had been filled in. The researchers contacted potential participants by telephone to give them more information about the study and to explain their participation, and then arranged a face-to-face meeting with them. 

The interviews were conducted by professionals with expertise in the ageing process and in working with older people. In view of the number of questionnaires, the interview sessions were approximately 30 min in length. All participants were given an informed consent form. 

#### 2.1.4. Data Analysis

The SPSS programme (version 26.0) was used for the analyses. Descriptive analyses of the sociodemographic variables were carried out. The relationships between the variables were analysed using Pearson correlations, after checking the normality of the data. 

### 2.2. Phase 2

#### 2.2.1. Participants

Once the results of Phase 1 had been obtained, potential focus group participants were contacted from among those who had taken part in Phase 1 and were willing to participate in this next phase of the study. The inclusion criteria were the same as in the previous phase, and no two people could be involved in the same focus group if they lived together and therefore provided care together. The researchers contacted 30 people from both centres. However, 11 of them declined the invitation, due to lack of time or health problems. In the end, 10 participants were recruited from the Guardamar del Segura Health Centre group and 9 from the Elda Health Centre group. 

#### 2.2.2. Data Collection

A focus group interview script was drawn up using the results of the first phase. The themes were as follows: (1) reason/Motivation for being a carer; (2) ambivalence of feelings; (3) managing personal space; (4) effects on health; (5) boundary between caring and parenting; (6) responsibility and decision making; (7) childcare tasks by sex.

#### 2.2.3. Procedure

Two focus groups were held between January and February 2022 in rooms set up for this purpose at the two health centres. The group sessions lasted approximately one to two hours. The groups were moderated by two researchers with expertise in qualitative research. One researcher facilitated the group discussion and asked questions, while the second researcher took field notes. Each topic was discussed individually until the participants arrived at a consensus. With the consent of the participants, the focus group discussions were video-recorded for later analysis. 

#### 2.2.4. Data Analysis

The validity of qualitative research is to be found both in the systematic process of recruiting subjects and in the analysis of the data that they provide [27] A six-stage thematic analysis was carried out in line with Braun’s approach [28]: (1) familiarisation with the data, (2) generation of initial categories or codes, (3) searching for themes, (4) reviewing themes, (5) defining and naming themes, and (6) producing the final report. Version 8 of the ATLAS.TI software was used.

### 2.3. Ethical Considerations

This study was approved by the University’s Bioethics Committee (Registration No. UA-2019-09-21) and the Drug Research Ethics Committee of the University Hospitals of Torrevieja, Elche-Vinalopó and Elda. It was conducted in accordance with the criteria established by the Declaration of Helsinki and the European Union’s Standards of Good Clinical Practice. All participants were informed by the researcher about the aim of the study, the methods used and the process for taking part. Written informed consent was obtained from the respondents prior to the interview.

## 3. Results

### 3.1. Phase 1

#### 3.1.1. Descriptive

Table 1 shows the results of the sociodemographic variables. In total, 100 participants aged from 54 to 77 years old (M = 67.26; SD = 4.10) took part. Of these, 68% of the sample were women and all were of Spanish nationality. A total of 88% were married or cohabiting, 10% were widowed, and 2% were separated or divorced. In terms of occupation, 70% were retired, 18% were homemakers, and 9% worked part time or full time. With respect to annual household income, 19% had an annual income of less than EUR 12,000, 75% had an income of EUR 12,000–29,999, and only 2% had an income of more than EUR 29,999. Of the total sample, 14% had not completed primary education, 69% had completed primary education, and 17% had completed secondary or higher education. 

The entire sample had grandchildren in their care, with a mean of 3.31 grandchildren (SD = 1.86, range = 1–9), and 80% had between one and four grandchildren. Six per cent reported sharing the care of their grandchildren. The majority of respondents (90%) were solely responsible for caring for between one and four grandchildren. Most often (43%), they cared for two grandchildren at the same time, spending an average of 24.53 h per week (SD = 17.30, range = 2–73) looking after only one grandchild and 12.40 h per week (SD = 12.93, range = 0–96) looking after two grandchildren at the same time. 

Table 2 shows the mean scores of the psychological variables assessed using the SF-12 Quality of Life Questionnaire, the abbreviated Yesavage Geriatric Depression Scale, and the Perceived Stress Scale (Cohen).

#### 3.1.2. Relationship between Variables

There is no association with stress or depressive symptoms for the variables number of hours of exclusive caring and number of years of caring (Table 3). 

Furthermore, there is no relationship between the mental health dimension of the SF-12 and the number of hours spent caring for grandchildren (*r* = 0.7, *p* = 0.06). However, there is a relationship between the number of hours of shared care of grandchildren and the depression score (*r* = 0.22, *p* < 0.05), the stress score (*r* = 0.22, *p* < 0.05) and the physical health dimension of the SF-12 (*r* = −0.29, *p* < 0.005). In other words, grandparents who share the care of their grandchildren more (compared to those who occasionally help their children with the care of their grandchildren) have increased symptoms of depression, greater stress, and poorer perceived physical health (health-related quality of life). 

### 3.2. Phase 2

Altogether, 19 grandparent carers took part. They were divided into two groups, with 10 participants in the Guardamar del Segura Health Centre group and 9 in the Acacias Health Centre group (Elda) (Table 4). 

Women accounted for 58% of the groups and men accounted for 42%. They ranged in age from 58 to 74 years old. Ninety per cent were married or co-habiting and ten per cent were widowed. None of the participants were single. Seventy-four per cent were in retirement and twenty-one per cent were in part-time or full-time employment. Between the two groups, they provided care for an average of 24 h per week. After reaching a broad consensus across the groups, the thematic analysis revealed two main themes and four sub-themes, as described in Table 5 below. 

#### 3.2.1. Broad Consensus: Positive Aspects

Caring for Grandchildren Helps You Stay Active:

Both groups agreed that caring for grandchildren forced them to be more active. This was generally seen as a good thing as they might otherwise be more sedentary:

“*We always say yes and never think about anything else, it helps us to stay active*”

PE3.

“*They make you move even if you don’t want to, you have to get up, they keep you active*”

PG7.

However, the complexity of the issue of grandparental care of grandchildren is highlighted, as both positive and negative aspects of such care are intertwined in the same sentence:

“*We’d be bored if it wasn’t for them () I admit that it’s more work, if you think about it sometimes you get very tired… but you do it willingly… but there’s nothing else for it, that’s just how it is*”

PE1.

This also reflects the influence of culture, the sense of familism and the paradox of perceiving positive aspects while pointing out the negative ones:

“*it’s all good, even though we have to get up early, but it’s our kids who need help, some days we don’t get home until 9 p.m. after picking them up at 5 p.m. and we don’t even have dinner or anything, we go straight to bed because we’re so tired, but what can you do? We have no choice () anyone would do it for their children*”

PG6.

#### 3.2.2. Broad Consensus: Negative Aspects

Loss of Personal and Leisure Time.

Respondents said that caring for their grandchildren deprived them of time for themselves. In this way, they acknowledged and agreed that caring for their grandchildren took up a lot of their free time and felt that they could not make decisions without first thinking about their grandchildren:

“*I have less time to go out into the countryside” PG5“you’re more restricted, because we have to rely on them (their children) for many things, for a trip away... in fact we’re going away now and I’m already worrying about how they’re going to cope” PE6 “they take it too far, they don’t ask if it’s alright, it’s as if you have no life of your own*”

PE2.

Excessive Responsibility.

The group participants spoke of feeling too much responsibility for the care of their grandchildren, as the time they had to spend with them meant that they had to get involved in their education and everyday lives. This caused them some discomfort, as they felt it was not their responsibility as grandparents:

“*we shoulder responsibilities that should not be ours at this age, yes, we do” PG9 “when you have them for so long, at some point you end up being a parent”PE4”I tell them there are things they can’t do at their grandparents’, even if they’re allowed to do it at home, and then my daughter tells me that yes they can do that at home (),there comes a point when you take on too much and you don’t know what to do*”

PG3.

Lack of Rest:

As with leisure time, participants in our study reported that they had to sacrifice their rest time in order to care for their grandchildren. Moreover, keeping up with their grandchildren’s schedule also forced them to modify their own sleep and rest patterns, which had a negative impact on both their physical and psychological perceived health.

“*I sleep less than I used to” PE9 “I get up earlier than when I was working” PG2 “at 6 o’clock I get up and they’re already here () and then I don’t have an afternoon nap because I have to go and pick them up” PE6 “If I didn’t take them, I wouldn’t be so tired, because you also have to take them to school, pick them up and then go to after-school activities, so you don’t get any time to rest*”

PG1.

Integration of approaches:

The integration of qualitative and quantitative data shows coherence as they explained how caring for grandchildren can lead to mental symptomatology due to lack of time for self, leisure and rest. On the other hand, the excess of responsibility they complained about during the focus groups would explain this symptomatology as well. The way of understanding the family and its role, in the grandparent carers’ words, would explain why these dynamics are maintained despite the grandparent carers’ perception of their health in relation to the care of their grandchildren.

## 4. Discussion

The aim of this study was to explore grandparents’ experiences of being involved in the upbringing and care of their grandchildren, and to determine how such activities affect their health-related quality of life. Together, the two methodological approaches show that shared care of grandchildren can lead to depressive symptoms and stress in grandparents when the obligatory nature and hours of care lead to an excess of responsibility in raising grandchildren and a lack of time for the grandparents’ own rest and personal lives. However, caring can help grandparents remain active and mobile. This could be seen as positive, were it not for the fact that it tends to lead to overexertion and time constraints for grandparent carers with negative repercussions on their perceived health-related quality of life. 

In terms of the profile of grandparent carers, our findings are partly consistent with studies on the same topic and in a nearby geographical context. They are also very similar in terms of the number of grandchildren being cared for and the time spent looking after them, and the ages of the grandchildren are also quite comparable [29]. 

In terms of the positive aspects shown by the qualitative analysis, these could be due to keeping grandparent carers active and on the go [30,31,32]. However, as both analyses show, this can become negative if caring becomes too burdensome for the grandparents, interfering with their need for rest and time for themselves and leading to excessive responsibility [33]. Some studies have also found that the perceived health of grandparent carers improved after they stopped caring for grandchildren [34]. Other authors only find positive health effects among grandmother carers [35] while some also suggest that caring for grandchildren does not have a causal effect on health-related quality of life [36]. At this point, it would be worth considering whether the qualitative focus group methodology may have led to participants feeling a certain need to mention positive aspects, despite highlighting negative ones, as a result of social desirability bias. 

At both the quantitative and qualitative psychological levels, increased levels of depressive symptoms are observed in the case of shared care. This is consistent with the results of studies such as that of McGarrigle et al. [37] in the case of intensive care or that of Komonpaisarn et al. [38], which does not report the intensity of care but does contradict the findings of studies showing that caring for grandchildren is a protective factor for maintaining mental health [30,32]. In this respect, the research shows conflicting results, with some reporting that depressive symptoms may increase when caring for grandchildren ceases [39], or even that caring for grandchildren is not associated with depressive symptoms [40] or that it has neither a positive nor negative influence on depression [41].

However, increased feelings of stress have been found both quantitatively and qualitatively when caring for grandchildren interferes with one’s rest and personal life and involves excessive responsibility, which is in line with the findings of Tang et al. [42] and Weisbrot y Giraudo [43] These findings contrast with those of Triadó et al. [44] who conclude that caring for grandchildren is a rewarding rather than a stressful situation regardless of its increasing intensity. 

These discrepancies and the heterogeneity in the results, as indicated in the literature, may be due to culture, how the concept of family is defined, the responsibilities traditionally assumed [42,45] or the hours dedicated to the care of grandchildren, the intensity of the care and whether it is carried out alone or on a shared basis [32,43]. 

We therefore agree with the conclusions of Chen and Liu [46], who state that caring does not lead to standardised positive or negative outcomes in the quality of life of carers but that this perception may depend on the form and intensity of the care provided and may also be conditioned by other factors at the personal and social level.

### Limitations and Strengths

To begin with, a convenience sample was drawn from two health centres. In order to be able to generalise the results, the study should be carried out in other centres with a nationally representative sample of grandparents. With regard to the qualitative study, it is worth noting that the sample consisted of only two focus groups; therefore, a larger sample may allow for a more complete understanding of grandparent carers. However, our sample does represent a variety of caring experiences at different times and with different ages and health situations, so our findings are transferable to other carers. Among the study’s strengths is its methodological complementarity. This helps us to make sense of the quantitative data via the voices of those involved. However, we believe that future lines of research could address the subject using in-depth interviews. Other lines of future research could include the following: (1) consider grandparents caring for their grandchildren in different cultural and socio-economic contexts; (2) research grandparents’ experiences of caring for their grandchildren at different ages and health conditions; (3) explore the impact of grandparents caring for their grandchildren on their long-term health and well-being; (4) examine how social and family policies that support grandparents in caring for their grandchildren can be designed and implemented.

## 5. Conclusions

Our mixed methods approach shows how caring for grandchildren can help grandparent carers stay active. However, this a priori positive aspect can become negative for their perceived quality of life when it comes to shared care and when grandparents’ willingness is abused. This may be due to their ingrained cultural values and lead to higher levels of stress and depression. However, further research is needed on the relationships between caring for grandchildren and grandparents’ health, as well as on the relationship between both grandparents’ and grandchildren’s health. 

## Figures and Tables

**Table 1:** Sociodemographic variables (N = 100).

		*N*
Gender	Women	68
Marital status	Married or civil partnership	88
	Separated or divorced	10
	Widowed	2
Nationality	Spanish	100
Employment status	Working full time	5
	Working part time	4
	Retired	70
	Unemployed	3
	Housewife	18
Annual wage	EUR <6000	1
	EUR 6000–8999	1
	EUR 9000–11,999	19
	EUR 12,000–17,999	43
	EUR 18,000–29,999	32
	EUR >30,000	2
Educational level	Incomplete primary education	14
	Complete compulsory studies	69
	Secondary education	14
	Higher education (undergraduate)	3
Number of grandchildren	1	14
	2	25
	3	23
	4	18
	>4	20
Range of hours of care	2–73 h	
Average hours of care	24.53 h	

**Table 2:** Mean scores of psychological variables.

		M (SD)
Depressive symptoms		1.68 (1.87)
Stress		13.53 (7.59)
Quality of life		
	Physical Health	46.88 (12.21)
	Mental Health	45.01 (7.50)

**Table 3:** Relationship between psychological variables and contact and/or care with grandchildren.

	Number of Hours Care	Years of Care	Number of Hours Shared Care
	r	r	r
Depressive symptoms	−0.15	−0.16	0.22 *
Stress	0.04	−0.04	0.22 *
Quality of life			
Physical Health	−0.05	0.03	−0.29 **
Mental Health	0.07	0.07	0.07

Note: calculated r Pearson correlations; * *p* < 0.05; ** *p* < 0.005.

**Table 4:** Sociodemographic data from the focus groups.

	Age	Gender	Marital Status	Employment Status	Nº of Children	Nº of Grandchildren *	Hours of Care **	Years of Care
**Guardamar del Segura Health Area Group**								
PG1	67	M	Married	Retired	3	5	45	3
PG2	69	M	Widowed	Retired	2	1	15	3
PG3	63	F	Married	Retired	2	3	30	11
PG4	66	M	Married	Retired	2	2	25	6
PG5	66	M	Married	Retired	2	3	6	6
PG6	69	F	Married	Retired	3	3	30	10
PG7	66	F	Married	Retired	2	3	10	6
PG8	66	F	Married	Working full time	3	4	40	4
PG9	74	M	Married	Retired	2	4	35	10
PG10	69	F	Married	Part-time work	2	3	40	14
**Elda Health Area Group**								
PE1	68	M	Married	Retired	2	5	15	11
PE2	68	F	Married	Retired	4	6	10	7
PE3	67	F	Married	Retired	2	2	30	14
PE4	72	F	Widowed	Retired	3	7	20	14
PE5	63	M	Married	Unemployed	2	2	10	7
PE6	58	F	Married	Working full time	2	2	15	4
PE7	67	F	Married	Retired	2	2	20	7
PE8	69	F	Married	Working full time	3	2	25	10
PE9	69	M	Married	Retired	2	3	30	12

* Number of grandchildren cared for; ** hours of care per week.

**Table 5:** Themes and sub-themes after analysis.

Grandparents’ Experiences of Care for Grandchildren	
Positive aspects	- Caring for grandchildren helps you stay active.
Negative aspects	- Loss of personal and leisure time; - Excessive responsibility; - Lack of rest.

## Data Availability

The data presented in this study are available on request from the corresponding author due to privacy and confidentiality issues.
